# Cognitive outcomes in chronic obstructive pulmonary disease (COPD)/OSA overlap syndrome compared to obstructive sleep apnea (OSA) alone: a systematic review

**DOI:** 10.1007/s11325-025-03426-9

**Published:** 2025-09-01

**Authors:** Ahmad M. Alharbi, Nawal Alotaibi, Ömer Faruk Uysal, Roby D. Rakhit, Simon E. Brill, John R. Hurst, Swapna Mandal

**Affiliations:** 1 Department of Respiratory Medicine, University College London, London, UK; 2 Institute of Cardiovascular Science, University College London, London, UK; 3 Cardiology Department, Royal Free London NHS Foundation Trust, London, UK; 4 Respiratory Department, Royal Free London NHS Foundation Trust, London, UK; 5 Prince Sultan Military College of Health Sciences, Dhahran, Saudi Arabia

**Keywords:** OSA, COPD, Overlap syndrome, Cognitive function, Nocturnal hypoxemia, Systematic review

## Abstract

**Background:**

Obstructive Sleep Apnoea (OSA) and Chronic Obstructive Pulmonary Disease (COPD) are both independently associated with cognitive impairment. COPD/OSA overlap syndrome could potentially result in greater cognitive impairment that is more than additive. This systematic review evaluates attention, memory, executive function and global cognition in OSA alone compared to COPD/OSA overlap syndrome.

**Methods:**

Systematic searches in MEDLINE, EMBASE, PsycINFO, CINAHL, and CENTRAL identified studies assessing cognitive function in adults with OSA and/or COPD/OSA overlap syndrome. Inclusion criteria required validated diagnostic and cognitive assessment tools. Twelve studies, including 7,424 participants, were reviewed: 10 involving OSA alone and 2 involving overlap syndrome. A narrative synthesis was performed due to methodological heterogeneity. Registration number is: CRD42024557577.

**Results:**

OSA alone was primarily associated with mild to moderate cognitive impairment, with attention and executive function most affected, with nocturnal hypoxemia and sleep fragmentation thought to be underlying causative factors. Memory and global cognition were relatively preserved. In contrast, COPD/OSA overlap syndrome was associated with more severe impairments, particularly in memory and global cognition. Overlap patients had significantly lower cognitive scores and a higher prevalence of mild cognitive impairment compared to OSA alone.

**Conclusions:**

Whilst OSA alone is associated with mild to moderate cognitive impairments, COPD/OSA overlap syndrome associates with more pronounced impairments, particularly in memory and global cognition. Nocturnal hypoxemia and systemic inflammation may be important mechanisms. Early cognitive screening and targeted interventions could support clinicians in mitigating these risks.

**Supplementary Information:**

The online version contains supplementary material available at 10.1007/s11325-025-03426-9.

## Introduction

Chronic Obstructive Pulmonary Disease (COPD) and Obstructive Sleep Apnea (OSA) are two of the most common respiratory conditions globally [1]. COPD is characterized by persistent airflow limitation and chronic inflammation, leading to hypoxemia and oxidative stress. OSA is marked by recurrent episodes of upper airway obstruction during sleep, causing intermittent hypoxemia, fragmented sleep, and sleep inefficiency [2]. OSA severity is classified based on the Apnea-Hypopnea Index (AHI), which measures the number of apneic episodes (complete pauses in airflow) and hypopneic episodes (partial reductions in airflow) per hour of sleep. The severity is categorized as mild (AHI 5–15 events/hour), moderate (AHI 15–30 events/hour), or severe (AHI > 30 events/hour) [3]. However, growing evidence highlighted that AHI alone does not adequately reflect the full clinical impact of OSA. The 2021 International Consensus on OSA recommends a broader approach that includes oxygen saturation patterns, sleep fragmentation, excessive daytime sleepiness, and comorbidities such as diabetes or obesity to more accurately determine the severity of OSA [4]. These factors collectively shape the overall hypoxic burden, which may be more closely associated with adverse clinical outcomes than AHI alone [5].

Both COPD and OSA have been strongly linked to cognitive impairments; particularly in attention, memory, executive function, and global cognition [6–8]. Data in those with OSA has consistently identified impairments in attention and executive function, which are attributed to hypoxemia and sleep fragmentation [7]. Similarly, COPD has demonstrated a strong association between chronic systemic inflammation and memory and attention [6].

COPD and OSA may coexist in one patient, forming what is referred to as COPD/OSA overlap syndrome, a concept that was first established in 1985 [9]. COPD/OSA overlap syndrome is associated with more severe nocturnal hypoxemia and associated therefore a higher risk of cognitive decline compared to COPD or OSA alone [10]. The pathophysiological mechanisms resulting from the combined effects of COPD and OSA may explain the higher risk of cognitive impairments in overlap syndrome. However, few studies have systematically compared cognitive outcomes in overlap syndrome compared with those who have OSA alone. Also, the specific cognitive domains most affected in COPD/OSA overlap syndrome compared to OSA alone and the severity of these impairments remain under explored. This gap in knowledge may limit clinicians’ ability to develop targeted interventions to mitigate cognitive decline in this high-risk population.

This systematic review aims to address two research questions:

What is the impact of COPD/OSA overlap syndrome on cognitive function compared to individuals with OSA alone?

Which cognitive domains are most affected in individuals with COPD/OSA overlap syndrome compared to OSA alone?

This systematic review synthesises findings from studies on cognitive outcomes in OSA and COPD/OSA overlap syndrome to identify the cognitive domains most affected, compares the severity of impairments, and explores the mechanisms contributing to these impairments. By clarifying these aspects, this review seeks to provide a foundation for future research as well as supporting clinicians in addressing the cognitive decline in these groups of patients.

A narrative synthesis approach was employed in this review due to the methodological heterogeneity of the included studies. Existing research varies significantly in terms of study designs, reported outcomes, and cognitive assessment tools. For instance, while some studies focus on global cognition using general screening tools such as the Montreal Cognitive Assessment (MoCA) and Mini-Mental State Examination (MMSE), others assess specific domains such as memory or executive function using distinct cognitive tests. Moreover, differences in population characteristics, such as the inclusion of younger versus older patients and the varying severity of hypoxemia, further complicate quantitative comparisons. By synthesizing findings narratively, the goal of this review is to provide a comprehensive understanding of cognitive impairments in COPD/OSA overlap syndrome compared to OSA alone, highlighting areas for future investigation.

## Methods

### Search strategy

A systematic search was conducted across MEDLINE, EMBASE, PsycINFO, CINAHL, and CENTRAL to identify relevant studies. The search strategy incorporated controlled vocabulary and keywords related to COPD; Chronic Obstructive Pulmonary Disease; Obstructive Sleep Apnea; OSA; Sleep-Disordered Breathing; Overlap Syndrome; Global cognition; Cognition; Neuropsychology; Cognitive Functioning; Memory; Attention; Executive Function; Processing Speed; Language/fluency; Visuospatial skills. This systematic review has been registered in PROSPERO (Registration ID: CRD42024557577).

### Types of included studies

Case-controlled studies and cross-sectional studies.

#### Inclusion criteria

Studies were included if they met the following criteria:

- Adults aged 18 years or older.
- Diagnoses of OSA or COPD-OSA overlap syndrome confirmed by validated sleep study and COPD confirmed by spirometry demonstrating post-bronchodilator FEV1/FVC < 0.70 (or below the lower limit of normal).
- Assessment of cognitive function using validated tools.
- Studies investigating the impact of cognitive function (such as memory, attention, executive function, processing speed, language/fluency, visuospatial skills), in COPD/OSA overlap syndrome, and OSA alone.

#### Exclusion criteria

- Non-English studies.
- Abstracts, case reports, narrative reviews, theses, books, conference proceedings, and self-reported diagnoses or physician diagnoses studies.
- Studies on non-human subjects.

### Data selection

**Fig. 1:**
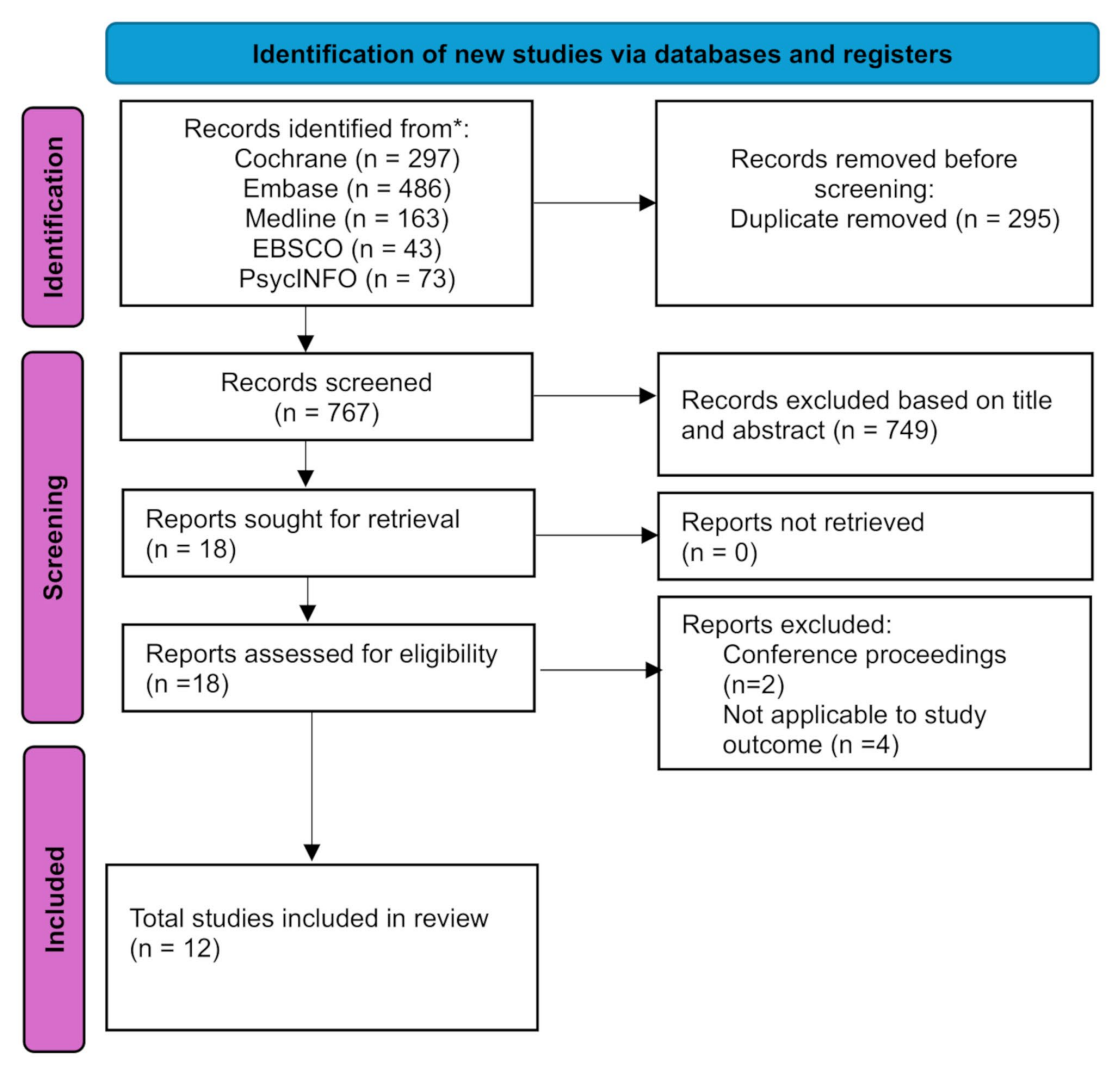
PRISMA Flow [11]

This review used (PRISMA-P 2015) in the process and the studies retrieved imported into EndNote software and duplicate studies were removed (Fig. 1). Two independent reviewers (AA&NA) blindly screened titles and abstracts of all articles using Rayyan based on the including criteria. Disagreements in screening were resolved through discussion or consultation with a third reviewer (SM). The studies included in this review were published between 2003 and 2024.

### Data extraction

- General information such as title, author, and date of publication.
- The study characteristics that include objectives and aims, study design, inclusion and exclusion criteria, participant demographic, and baseline characteristics, primary and secondary outcomes related to cognitive function.
- The criteria used to diagnose OSA and COPD/OSA overlap syndrome.

### Quality assessment

The Newcastle-Ottawa Scale (NOS) was used to evaluate the methodological quality of included studies. Studies were scored on selection, comparability, and outcome assessment, with scores ranging from 0 to 10.

### Data synthesis

A narrative synthesis was employed to analyse the findings due to the heterogeneity in study designs and outcome measures. Key cognitive domains such as attention, memory, executive function was analysed separately to identify patterns and severity of impairments. Comparisons between OSA and COPD-OSA overlap syndrome were emphasized, focusing on cognitive domains, severity, and underlying mechanisms.

## Results

This paper explores cognitive impairments associated with Obstructive Sleep Apnea (OSA) alone and overlap syndrome (COPD/OSA). The results are categorized into cognitive domains: attention, memory, executive function, and global cognition, focusing on severity and mechanisms. These findings are drawn from 12 studies encompassing 7,424 participants, with 10 studies focusing on OSA alone (7,289 participants) and 2 studies addressing the overlap of COPD and OSA (135 participants).

### Study characteristics

The included studies represent diverse populations and methodologies, mostly cross-sectional designs. Cognitive outcomes were assessed using a variety of validated tools, including domain-specific tests such as Stroop Test, Trail Making Test (TMT), and Digit Span and global measures such as the Montreal Cognitive Assessment (MoCA) and Mini-Mental State Examination (MMSE). The characteristics of all included studies are summarized in Table 1.

**Table 1:** Studies characteristics

Authors		Population	Sample Size	Mean Age (Years)	AHI (Events/h)	Diagnostic Method Used	Gender Distribution. Male/Female	Healthy/Control Group^1^	Key Cognitive Tools^2^	Focus
Shpirer et al.2012 [7]		OSA Alone	*N* = 40	53.3 ± 11.1	*n* = 11 patients (AHI ≤ 15) *n* = 15 patients (15 < AHI ≤ 30) *n* = 14 patients (AHI > 30)	Full in-lab polysomnography (PSG)	32 / 8	NO	Conners’ Test (CPT), Trail Making Test (TMT A and B), Digit Span subtest, Tower of London (TOL), Wisconsin Card Sorting Test (WCST),and Verbal Fluency Test.	Attention and executive function.
Giora et al. 2017 [12]		OSA Alone	*N* = 38	45.5 ± 13.0	OSA: 47.87 (± 24.67)	Type 3 Polygraphy	25 / 13	*n* = 19	Visual search paradigm using matrixes of letters to identify a target (‘T’) among distractors.	Visual perception, Accuracy in a visual search task.
Antonelli et al. 2004 [13]		OSA Alone	*N* = 49	61.5	NA	Type 3 Polygraphy	NA	NO	Mental Deterioration Battery	Thinking, verbal attainment, and constructive ability.
Pase et al. 2023 [14]		OSA Alone	*N* = 5946	59.4 ± 12.0	AHI > 5	Home PSG (Type II)	4071 / 1875	NO	MMSE	Executive function., attention and processing speed, verbal learning, memory, language, and visuospatial function.
Ningzhen et al. 2019 [15]		OSA Alone	*N* = 134	37.54 ± 7.66	AHI < 5 (*n* = 25) AHI 5–14.9 (*n* = 28) AHI 15–30 (*n* = 26) AHI > 30 (*n* = 55)	Full in-lab PSG	NA	NO	MoCA	Visual-spatial ability, executive function, memory, attention, language, abstraction, delayed recall, andorientation.
Sforza et al. 2010 [8]		OSA Alone	*N* = 445	68 (± 1.8)	30.35 ± 12.7	Type 3 Polygraphy	41.5% / 58.5%	NO	MMSE	Executive function, attention and processing speed, verbal learning, memory, language, andvisuospatial function.
Hong et al. 2024 [16]		OSA Alone	*N* = 102	37.54 ± 7.66	*n* = 37 (AHI < 15) *n* = 65 (AHI ≥ 15).	Full in-lab PSG	NA	NO	MoCA, Stroop Color, Word Test (SCWT), and Digit Span Test (DST).	Visual-spatial ability, executive function, memory, attention, language, abstraction, delayed recall, and orientation.
Naismith et al. 2004 [17]		OSA Alone	*N* = 100	50.3 ± 9.6	26.3 ± 17.4	Full in-lab PSG	76 / 14	NO	Trail Making Test, Symbol Digit Modalities Test, Digit Span subtest, Rey Auditory Verbal Learning Test, Verbal and Semantic Fluency tests, Vocabulary subtest, Block Design subtest, Wisconsin Card Sorting Test, and Tower of London.	Executive function, attention and processing speed, verbal learning, memory, language, and visuospatial function.
Verstraeten et al. 2004 [18]		OSA Alone	*N* = 68	48.3 ± 7.8	60.5 ± 31.6	Full in-lab PSG	46 / 22	*n* = 32	Trail Making Test (TMT) A and B, Symbol Digit Modalities Test (SDMT), Digit Span Test (forward and backward), Stroop Color-Word Test, Five-Point Test (figural fluency), andFlexibility Task (attention switching).	Attention and executive function
Daniel et al. 2003 [19]		OSA Alone	*N* = 508	79–97 years old	*n* = 376 (AHI 5–29) *n* = 132 (AHI > 30)	Home PSG	508 / -	NO	Cognitive Abilities Screening Instrument (CASI)	Attention, concentration, and memory.
Wang et al. 2020 [20]		COPD/OSA	*N* = 106	67.7 ± 10.6	35 (27, 44)	Full in-lab PSG	90 / 16	NO	MoCA	Visual-spatial ability, executive function, memory, attention, language, abstraction, delayed recall, and orientation.
Zhang et al. 2020 [21]		COPD/OSA	*N* = 29	70.4 ± 7.8	29.3 ± 11.4	Full in-lab PSG	24 / 5	NO	MMSE	Executive function, attention and processing speed, verbal learning, memory, language, andvisuospatial function.

o AHI = Apnea-Hypopnea Index; MoCA = Montreal Cognitive Assessment; MMSE = Mini-Mental State Examination

o N represents the total sample size of the study

o n represents the number of participants in specific subgroups

o ^1^Healthy/Control Group indicates whether a comparative healthy population was included in the study

o ^2^Key Cognitive Tools are instruments used for assessing specific cognitive domains

### Cognitive outcomes in obstructive sleep apnea alone (OSA)

#### Attention domain

Impairments in the attention domain in OSA were reported across five studies, with study sample sizes ranging from 40 to 5,946 participants. The severity of attention impairments ranged from mild to moderate, with differing mechanisms identified. Shpirer et al. (2012) identified attention as the most affected cognitive domain, with performance significantly correlated with both OSA severity (AHI: *r* = 0.6, *p* < 0.001) and hypoxemia measures, including average oxygen saturation (*r* = − 0.51, *p* = 0.002) and percent time with SpO_2_ < 90% (*r* = 0.57, *p* < 0.001) [7]. Hong et al. (2024) reported slower Stroop Test completion times in moderate-to-severe OSA, though attention sub-scores between OSA severity groups were not significantly different (*p* = 0.068) [16]. Pase et al. (2023) observed moderate impairments in attention-related tasks were associated with higher AHI and oxygen desaturation index (ODI) [14]. However, Verstraeten et al. (2004) and Sforza et al. (2010) reported milder impairments in attention-related tasks, attributed to sleep fragmentation and age-related factors [8, 18]. A summary of attention domain findings is presented in Table 2.

**Table 2:** Attention domain key findings among OSA patients

Category	Authors	Key Findings	Severity of attention impairments	Mechanism
OSA	Shpirer et al. (2012), Sforza et al. (2010), Hong et al. (2024), Pase et al. (2023), and Verstraeten et al. (2004)	• Significant correlation between attention impairments and AHI and hypoxemia. • REM sleep linked to better attention performance in OSA patients. • Attention deficit was linked to sleep fragmentation.	Mild to moderate	Nocturnal hypoxemia; REM sleep disruption, and sleep fragmentation.

o AHI: Apnea-Hypopnea Index, CPT: Continuous Performance Test, MoCA: Montreal Cognitive Assessment, REM sleep: Rapid Eye Movement sleep

#### Executive function domain

Three studies reported impairments in executive function in OSA; sample sizes ranged from 40 to 68 participants, and the severity of executive function impairments varied from mild to moderate. Verstraeten et al. (2004) found no evidence of significant executive function decline in OSA patients and attributed mild impairments on the Flexibility Test to reduced attentional capacity rather than executive dysfunction. The study concluded that executive function deficits are not a major concern in OSA alone and are secondary to attentional disruptions caused by sleep fragmentation [18]. In contrast, Shpirer et al. (2012) reported moderate impairments in planning, cognitive flexibility, and verbal fluency, demonstrated by deficits in tasks such as the Wisconsin Card Sorting Test (*p* < 0.001) and Tower of London (*p* = 0.04). While no significant correlations were found between these impairments and AHI or hypoxemia, hypoxemia was noted as a potential contributing factor due to its known effects on oxygen-sensitive brain regions critical to executive functioning [7]. Antonelli Incalzi et al. (2004) similarly reported moderate executive dysfunction, with OSA patients showing low scores on tasks such as analogies and temporal rule induction and were linked to the combined effects of sleep fragmentation and intermittent hypoxia on frontal-lobe structures [13]. A summary of findings related to executive function is provided in Table 3.

**Table 3:** Executive function domain key findings among OSA patients

Category	Authors	Key Findings	Severity of executive function impairments	Mechanism
OSA	Shpirer et al. (2012), Verstraeten et al. (2004), and Antonelli Incalzi et al. (2004)	• Mild to Moderate impairments in deductive thinking, planning, cognitive flexibility, and verbal fluency influenced by daytime sleepiness, sleep fragmentation, and hypoxemia	Mild to Moderate	Sleep fragmentation, daytime sleepiness and hypoxemia

o TMT-B: Trail Making Test Part B. WCST: Wisconsin Card Sorting Test. TOL: Tower of London

#### Memory domain

Memory deficits in OSA were assessed in three studies, involving a total of 6,552 participants, with individual sample sizes ranging from 100 to 5,946. The findings consistently indicated minimal impact of OSA on this cognitive domain. Naismith et al. (2004) used the Digit Span subtest and Rey Auditory Verbal Learning Test (RAVLT) and found no significant associations between memory performance and hypoxemia or sleep fragmentation [17]. Similarly, Foley et al. (2003) observed no relationship between OSA and short-term memory in elderly Japanese American men, as assessed by the CASI short-term memory subcomponent [19]. Pase et al. (2023), analysing five large datasets, reported no significant associations between OSA severity and memory scores. These findings consistently suggest that memory is less affected by OSA compared to domains like attention or executive function, with no strong associations observed with hypoxemia, sleep fragmentation, or other OSA-related factors [14]. A summary of findings related to memory outcomes is provided in Table 4.

**Table 4:** Memory domain key findings among OSA patients

Category	Authors	Key Findings	Severity of memory impairments	Mechanism
OSA	Naismith et al. (2004), Foley et al. (2003), and Pase et al. (2023)	• No significant association between memory performance and sleep indices (AHI, hypoxemia). • Memory relatively unaffected in all studies.	None to minimal impact.	No strong link to OSA-related factors

o Digit Span: Test for working memory capacity. RAVLT: Rey Auditory Verbal Learning Test. CASI: Cognitive Abilities Screening Instrument. AHI: Apnea-Hypopnea Index

### Global cognition

Three studies assessed global cognitive function in OSA, involving 1,052 participants with sample sizes ranging from 102 to 445. Findings varied based on population characteristics and severity of OSA. Li et al. (2019) found a statistically significant decline in MoCA scores with increasing OSA severity, with mild OSA patients scoring the highest (mean MoCA score: 26.0 ± 2.0) compared to those with moderate to severe (25.0 ± 2.0 and 25.5 ± 2.0, respectively) (*p* < 0.01). Li et al. (2019) found a negative correlation between sleep fragmentation, as measured by the phase A3 index of cyclic alternating patterns (CAP phase A3) and MoCA scores (*r* = -0.329, *p* < 0.01) [15]. Sforza et al. (2010) observed no significant associations between MMSE scores and AHI or oxygen desaturation index in an elderly population, suggesting that global cognitive impairments in older adults are limited and more influenced by other age-related factors [8]. Hong et al. (2024) reported significantly lower MoCA scores in moderate-to-severe OSA patients compared to mild OSA or healthy controls (25.0 vs. 28.0, *p* < 0.001). Specific impaired MoCA domains included: visuospatial and executive abilities, delayed recall, and language, with strong negative correlations between MoCA scores and AHI (*r* = -0.481, *p* < 0.001), sleep inefficiency (*r* = -0.441, *p* < 0.001), and hypoxemia markers (*r* = -0.444, *p* < 0.001) [16]. A summary of findings related to global cognitive function is presented in Table 5.

**Table 5:** Global cognition domain key findings among OSA patients

Category	Authors	Key Findings	Severity of global cognition impairments	Mechanism
OSA	Li et al. (2019), Sforza et al. (2010), and Hong et al. (2024)	Moderate-severe OSA group scored lower on MoCA with correlations to AHI, sleep inefficiency, and hypoxemia	Mild to moderate.	• Sleep fragmentation • Hypoxemia • AHI

• MoCA: Montreal Cognitive Assessment. MMSE: Mini-Mental State Examination

### Cognitive outcomes in COPD/OSA overlap syndrome

Only two studies have specifically investigated cognitive outcomes in COPD/OSA overlap syndrome. Wang et al. (2020) and Zhang et al. (2020) reported significant cognitive impairments in patients with COPD/OSA overlap syndrome, based on 135 participants, with individual study sizes of 106 and 29 respectively. Wang et al. (2020) found moderate to severe impairments. MoCA attention subtest scores were lower in overlap patients compared to COPD-only patients (24.0 vs. 26.0, *p* < 0.001), associated with nocturnal hypoxemia (percentage of time spent with oxygen saturation below 90% (TSat90), 2.54% vs. 0.59%, *p* < 0.001). Memory impairments were classified as severe because overlap patients exhibited significantly worse MMSE scores (23.5 vs. 25.5, *p* = 0.01) with higher rates of mild cognitive impairment (MCI, 40.6% vs. 24.6%, *p* = 0.005). Hypoxemia was the strongest predictor of memory decline in COPD/OSA overlap syndrome (OR = 4.75, 95% CI: 2.73–11.13, *p* < 0.001). Executive function impairments were classified as moderate; Wang et al. (2020) highlighted abstraction and problem-solving deficits on MMSE subtests. Zhang et al. (2020) linked intermittent hypoxia (OR = 1.24, 95% CI: 1.04–1.48, *p* = 0.02) to global cognitive decline. Global cognition was significantly impaired in overlap syndrome patients. Zhang et al. (2020) reporting that 66% of overlap patients were at risk of dementia (MMSE ≤ 24, *p* < 0.01) [20, 21]. A summary of findings related to cognitive outcomes in COPD/OSA overlap syndrome is provided in Table 6.

**Table 6:** Cognitive domains key findings among COPD/OSA patients

Cognitive Domain	Key Findings	Severity	Mechanism	Authors
Attention	- Moderate to severe impairments in MoCA attention subtests (24.0 vs. 26.0, *p* < 0.001). - Linked to nocturnal hypoxemia (TSat90, 2.54% vs. 0.59%, *p* < 0.001).	Moderate to severe	Nocturnal hypoxemia (TSat90).	Wang et al. (2020) and Zhang et al. (2020)
Memory	- Severe impairments with significantly worse MMSE scores (23.5 vs. 25.5, *p* = 0.01). - Higher rates of MCI in overlap patients (40.6% vs. 24.6%, *p* = 0.005). - Hypoxemia as the strongest predictor (OR = 4.75, *p* < 0.001).	Severe	Nocturnal hypoxemia	
Executive Function	- Moderate impairments in abstraction and problem-solving tasks on MMSE. - Linked to intermittent hypoxia (OR = 1.24, 95% CI: 1.04–1.48, *p* = 0.02).	Moderate	Intermittent hypoxia	
Global Cognition	- Significantly impaired global cognition, with 66% of overlap patients at risk of dementia (MMSE ≤ 24, *p* < 0.01).	Moderate to severe	Nocturnal hypoxemia	

• MoCA: Montreal Cognitive Assessment. MMSE: Mini-Mental State Examination. MCI: Mild Cognitive Impairment

• TSat90: Percentage of time spent with oxygen saturation below 90%

• OR: Odds Ratio (used in regression analysis)

• 95% CI: Confidence Interval, indicating the range within which the true effect lies with 95% confidence

• *p*-value: Probability value indicating the statistical significance of the result (*p* < 0.05 is considered significant)

## Discussion

### Overview of findings

This review systematically evaluated the cognitive impairments associated with OSA alone and COPD/OSA overlap syndrome across key cognitive domains, including attention, memory, executive function, and global cognition. Across these domains, the data demonstrated over mild to moderate impact on cognitive function in those with OSA alone. Whilst COPD/OSA overlap syndrome was associated with more severe cognitive impairments in comparison, particularly in memory and global cognition and primarily caused by nocturnal hypoxemia and systemic inflammation.

### Cognitive function in OSA and COPD/OSA overlap syndrome

In OSA, cognitive impairments were mostly limited to domains dependent on the prefrontal cortex, such as attention and executive function. Several studies have shown that effective performance on tests requiring planning and attention depends on proper functioning of the prefrontal cortex [22–24]. Intermittent hypoxemia and sleep fragmentation are known to disrupt sleep architecture and induce oxidative stress in OSA that ultimately affect prefrontal cortex function [25]. These mechanisms align with observed impairment in sustained attention and planning tasks as reported in several studies [7, 8, 13, 14, 16, 18]. However, memory and global cognition tend to remain relatively preserved among OSA patients [14, 17, 19]. One possible explanation is that the hippocampus, the essential structure for memory, may be less impacted in OSA [26, 27]. The hippocampus may be partially recovered during periods of normoxia, particularly when OSA is less severe or is effectively treated with CPAP [28]. Moreover, patients’ differences in age and comorbidities further influence cognitive outcomes. Finally, since memory is a fundamental component of global cognition, any preservation of memory processes may help maintain global cognitive performance. This overlap in memory and global cognition may help explain why both memory and global cognitive abilities often show similar patterns of relative preservation in some OSA patients [27].

In COPD/OSA overlap syndrome, cognitive impairments are more pronounced compared to OSA alone, particularly in memory and global cognition [20, 21]. The high effect on cognitive is likely due to the compounded effects of sustained hypoxemia from COPD and intermittent hypoxemia from OSA, which together can place a greater physiological burden on the brain [21, 29, 30]. Prolonged hypoxemia can lead to neuronal damage through mechanisms such as oxidative stress, systemic inflammation, and endothelial dysfunction, which collectively impair cognitive function across multiple cognitive domains [21, 31].

Memory impairments, while observed in OSA, tend to be less severe compared to other cognitive domains such as attention and executive function [7, 8, 13, 14, 16–19]. In contrast, memory difficulties are more pronounced in overlap syndrome, likely due to the compounded effects of sustained and intermittent hypoxemia. Studies have shown that COPD/OSA overlap syndrome results in more severe and sustained hypoxemia compared to OSA alone, as the chronic respiratory compromise in COPD exacerbates the oxygen desaturation caused by OSA [32, 33]. This high burden of hypoxemia may cause higher risk of neuronal injury, especially to the hippocampus, leading to deficits in memory encoding and retrieval [26, 34]. Furthermore, systemic inflammation, a hallmark of COPD, exacerbates neuronal injury, particularly in brain regions involved in memory and global cognition [20, 21, 32, 35].

### Strengths and limitations

This narrative synthesis approach offering valuable insights into the differential cognitive impacts of OSA and COPD/OSA overlap syndrome [36]. However, there are several limitations identified. First, the heterogeneity in cognitive assessment tools restricted the direct comparison across studies. Second, the variability in age and disease severity within study populations limited the generalizability of findings. Third, it is unclear whether patients in the included studies were treated with continuous positive airway pressure (CPAP), which may have influenced cognitive outcome. Forth, although all included studies used either full polysomnography or cardiorespiratory polygraphy, variation in monitoring protocols such as home-based versus in-lab assessments may introduce differences in diagnostic precision and sleep architecture assessment. Fifth, many of the included studies featured predominantly male participants, which may reduce the generalizability of findings to female populations. Sixth, all studies were cross-sectional or case-control, restricting causal interpretation and insights into long-term cognitive changes. Seventh, restricting the review to English-language publications may introduce language bias and exclude relevant studies in other languages. Eighth, the small number of studies focusing on overlap syndrome created an imbalance in the evidence base, restricting the ability to draw robust conclusions for this group. Longitudinal studies are essential to evaluate the progression of cognitive decline, particularly in overlap syndrome patients, and to strengthen the evidence base for targeted interventions. Also, future research should aim for standardized testing protocols across populations to enable robust meta-analyses.

### Clinical implications

The systematic review findings highlight the importance of early cognitive screening in both OSA and COPD/OSA overlap syndrome patients. Clinicians can incorporate standardized cognitive assessment tools, such as MoCA or MMSE, as part of routine clinical evaluations, particularly for patients with severe nocturnal hypoxemia, frequent exacerbations, or diagnosed overlap syndrome, which place them at higher risk of cognitive decline. Also, a brief, domain-specific tests, such as TMT for executive function or the Stroop Test for attention can be integrated to help identify cognitive impairments early. These tests can be incorporated into outpatient respiratory or sleep clinics when discussing disease management. Additionally, CPAP therapy is an established treatment for OSA and has been shown to reduce nocturnal hypoxemia [37–39]. Several studies indicated that CPAP may partially improve cognitive impairments in OSA patients, particularly in attention, memory, and executive function [38–43]. In COPD/OSA overlap syndrome, CPAP has been found to reduce exacerbations, hospitalizations, and mortality [44, 45]. However, its direct impact on cognitive function in overlap syndrome remains unclear, as research specifically addressing this question is still limited. Further studies are needed to better understand cognitive outcomes in patients with COPD/OSA overlap syndrome and to evaluate whether interventions like CPAP can help prevent or improve cognitive impairment. Moreover, future research should incorporate detailed measures of hypoxic burden, including the depth and duration of oxygen desaturation, to better capture the physiological stress associated with OSA. These measures may offer greater clinical relevance than AHI alone, particularly when examining associations with cognitive and systemic health outcomes [5]. Beyond clinical application, future research should emphasize the use of standardized cognitive assessment tools and comprehensive statistical reporting including group means, standard deviations, and control comparisons to enhance cross-study comparability and facilitate robust meta-analytic synthesis.

## Conclusion

This review highlights the differential cognitive impacts of OSA and COPD/OSA overlap syndrome. Whilst OSA alone is associated with mild to moderate cognitive impairments, COPD/OSA overlap syndrome associates with more pronounced impairments, particularly in memory and global cognition. Nocturnal hypoxemia and systemic inflammation may be important mechanisms but further work to understanding the underlying pathophysiology is required. Early cognitive screening and targeted interventions could support clinicians in mitigating these risks and should be incorporated into clinical pathways.

## Electronic supplementary material

Below is the link to the electronic supplementary material.

Supplementary Material 1

Supplementary Material 2

Supplementary Material 3

Supplementary Material 4

Supplementary Material 5

Supplementary Material 6

Supplementary Material 7

## Data Availability

This study is a systematic review of previously published research. All data analysed in this review are derived from publicly available studies that are cited within the manuscript. No new data were generated or collected for this study.

## References

[1] Czerwaty K, Dżaman K, Sobczyk KM, Sikorska KI (2023) The overlap syndrome of obstructive sleep apnea and chronic obstructive pulmonary disease: A systematic review. Biomedicines 11(1):16 10.3390/biomedicines11010016 PMC9856172 36672523

[2] Global Initiative for Chronic Obstructive Lung Disease - GOLD [cited 2025 May 19]. 2025 GOLD Report. Available from: https://goldcopd.org/2025-gold-report/

[3] Soori R, Baikunje N, D’sa I, Bhushan N, Nagabhushana B, Hosmane GB (2022) Pitfalls of AHI system of severity grading in obstructive sleep Apnoea. Sleep Sci 15(Spec 1):285–288 35273779 10.5935/1984-0063.20220001 PMC8889969

[4] Chang JL, Goldberg AN, Alt JA, Alzoubaidi M, Ashbrook L, Auckley D et al (2023) International consensus statement on obstructive sleep apnea. Int Forum Allergy Rhinol 13(7):1061–1482 36068685 10.1002/alr.23079 PMC10359192

[5] Azarbarzin A, Sands SA, Stone KL, Taranto-Montemurro L, Messineo L, Terrill PI et al (2019) The hypoxic burden of sleep Apnoea predicts cardiovascular disease-related mortality: the osteoporotic fractures in men study and the sleep heart health study. Eur Heart J 40(14):1149–1157 30376054 10.1093/eurheartj/ehy624 PMC6451769

[6] Torres-Sánchez I, Rodríguez-Alzueta E, Cabrera-Martos I, López-Torres I, Moreno-Ramírez MP, Valenza MC (2015) Cognitive impairment in COPD: a systematic review. J Bras Pneumol 41(2):182–190 25909154 10.1590/S1806-37132015000004424 PMC4428856

[7] Shpirer I, Elizur A, Shorer R, Peretz RB, Rabey JM, Khaigrekht M (2012) Hypoxemia correlates with attentional dysfunction in patients with obstructive sleep apnea. Sleep Breath Schlaf Atm 16(3):821–827 10.1007/s11325-011-0582-1 21898098

[8] Sforza E, Roche F, Thomas-Anterion C, Kerleroux J, Beauchet O, Celle S et al (2010) Cognitive function and sleep related breathing disorders in a healthy elderly population: the synapse study. Sleep 33(4):515 20394321 10.1093/sleep/33.4.515 PMC2849791

[9] Shah AJ, Quek E, Alqahtani JS, Hurst JR, Mandal S (2022) Cardiovascular outcomes in patients with COPD-OSA overlap syndrome: A systematic review and meta-analysis. Sleep Med Rev 63:101627 35413500 10.1016/j.smrv.2022.101627

[10] Luehrs RE, Moreau KL, Pierce GL, Wamboldt F, Aloia M, Weinberger HD et al (2021) Cognitive performance is lower among individuals with overlap syndrome than in individuals with COPD or obstructive sleep apnea alone: association with carotid artery stiffness. J Appl Physiol 131(1):131–141 33982592 10.1152/japplphysiol.00477.2020 PMC8325616

[11] Page MJ, McKenzie JE, Bossuyt PM, Boutron I, Hoffmann TC, Mulrow CD et al (2021) The PRISMA 2020 statement: an updated guideline for reporting systematic reviews. BMJ 372:n71 33782057 10.1136/bmj.n71 PMC8005924

[12] Giora E, Galbiati A, Marelli S, Zucconi M, Ferini-Strambi L (2017) Evidence of perceptive impairment in OSA patients investigated by means of a visual search task. Cortex J Devoted Study Nerv Syst Behav 95:136–142 10.1016/j.cortex.2017.08.004 28869823

[13] Antonelli Incalzi R, Marra C, Salvigni BL, Petrone A, Gemma A, Selvaggio D et al (2004) Does cognitive dysfunction conform to a distinctive pattern in obstructive sleep apnea syndrome? J Sleep Res 13(1):79–86 14996039 10.1111/j.1365-2869.2004.00389.x

[14] Pase MP, Harrison S, Misialek JR, Kline CE, Cavuoto M, Baril AA et al (2023) Sleep architecture, obstructive sleep apnea, and cognitive function in adults. JAMA Netw Open 6(7):e2325152 37462968 10.1001/jamanetworkopen.2023.25152 PMC10354680

[15] Li N, Wang J, Wang D, Wang Q, Han F, Jyothi K et al (2019) Correlation of sleep microstructure with daytime sleepiness and cognitive function in young and middle-aged adults with obstructive sleep apnea syndrome. Eur arch Oto-Rhino-Laryngol off J Eur fed Oto-Rhino-Laryngol Soc EUFOS affil Ger Soc Oto-Rhino-Laryngol -. Head Neck Surg 276(12):3525–3532 10.1007/s00405-019-05529-y 31263979

[16] Hong Y, Pei C, Hao L, Xu K, Liu F, Ding Z (2024) The study of the relationship between moderate to severe sleep obstructive apnea and cognitive impairment, anxiety, and depression. Front Neurol 15:1363005 38798707 10.3389/fneur.2024.1363005 PMC11119744

[17] Naismith S, Winter V, Gotsopoulos H, Hickie I, Cistulli P (2004) Neurobehavioral functioning in obstructive sleep apnea: differential effects of sleep quality, hypoxemia and subjective sleepiness. J Clin Exp Neuropsychol 26(1):43–54 14972693 10.1076/jcen.26.1.43.23929

[18] Verstraeten E, Cluydts R, Pevernagie D, Hoffmann G (2004) Executive function in sleep apnea: controlling for attentional capacity in assessing executive attention. Sleep 27(4):685–693 15283003

[19] Foley DJ, Masaki K, White L, Larkin EK, Monjan A, Redline S (2003) Sleep-disordered breathing and cognitive impairment in elderly Japanese-American men. Sleep 26(5):596–599 12938814 10.1093/sleep/26.5.596

[20] Wang Y, Li B, Li P, Gong T, Wu M, Fu J et al (2020) Severe obstructive sleep apnea in patients with chronic obstructive pulmonary disease is associated with an increased prevalence of mild cognitive impairment. Sleep Med 75:522–530 32828695 10.1016/j.sleep.2020.05.002

[21] Zhang XL, Gao B, Han T, Xiang BY, Liu X (2020) Moderate-to-Severe obstructive sleep apnea and cognitive function impairment in patients with COPD. Int J Chron Obstruct Pulmon Dis 15:1813 32801679 10.2147/COPD.S257796 PMC7396955

[22] Goldberg E (2001) The executive brain: frontal lobes and the Civilized Mind. Oxford University Press, p 276

[23] Fuster JM (1999) Synopsis of function and dysfunction of the frontal lobe. Acta Psychiatr Scand 99(s395):51–57 10225333 10.1111/j.1600-0447.1999.tb05983.x

[24] Barkley RA (2000) Genetics of childhood disorders: XVII. ADHD, part 1: the executive functions and ADHD. J Am Acad Child Adolesc Psychiatry 39(8):1064–1068 10939238 10.1097/00004583-200008000-00025

[25] Beebe DW, Gozal D (2002) Obstructive sleep apnea and the prefrontal cortex: towards a comprehensive model linking nocturnal upper airway obstruction to daytime cognitive and behavioral deficits. J Sleep Res 11(1):1–16 10.1046/j.1365-2869.2002.00289.x 11869421

[26] Fortin NJ, Agster KL, Eichenbaum HB (2002) Critical role of the hippocampus in memory for sequences of events. Nat Neurosci 5(5):458–462 10.1038/nn834 PMC4053170 11976705

[27] Ghetti S, Bunge SA (2012) Neural changes underlying the development of episodic memory during middle childhood. Dev Cogn Neurosci 2(4):381–395 10.1016/j.dcn.2012.05.002 PMC3545705 22770728

[28] Rosenzweig I, Glasser M, Polsek D, Leschziner GD, Williams SCR, Morrell MJ (2015) Sleep Apnoea and the brain: a complex relationship. Lancet Respir Med 3(5):404–414 25887982 10.1016/S2213-2600(15)00090-9

[29] Yaffe K, Laffan AM, Harrison SL, Redline S, Spira AP, Ensrud KE et al (2011) Sleep-disordered breathing, hypoxia, and risk of mild cognitive impairment and dementia in older women. JAMA 306(6):613–619 10.1001/jama.2011.1115 PMC3600944 21828324

[30] Dodd JW, Getov SV, Jones PW (2010) Cognitive function in COPD. Eur Respir J 35(4):913–922 10.1183/09031936.00125109 20356988

[31] Dash UC, Bhol NK, Swain SK, Samal RR, Nayak PK, Raina V et al (2024) Oxidative stress and inflammation in the pathogenesis of neurological disorders: Mechanisms and implications. Acta Pharm Sin B. Oct 16 [cited 2025 Jan 27]; Available from: https://www.sciencedirect.com/science/article/pii/S2211383524004040 10.1016/j.apsb.2024.10.004 PMC11873663 40041912

[32] Budhiraja R, Siddiqi TA, Quan SF (2015) Sleep disorders in chronic obstructive pulmonary disease: etiology, impact, and management. J Clin Sleep Med 11(3):259–270 10.5664/jcsm.4540 PMC4346647 25700872

[33] McNicholas WT (2019) Does associated chronic obstructive pulmonary disease increase morbidity and mortality in obstructive sleep apnea?? Ann Am Thorac Soc 16(1):50–53 30592452 10.1513/AnnalsATS.201809-628ED

[34] Borson S, Scanlan J, Friedman S, Zuhr E, Fields J, Aylward E et al (2008) Modeling the impact of COPD on the brain. Int J Chron Obstruct Pulmon Dis 3(3):429–434 10.2147/copd.s2066 PMC2629981 18990971

[35] Lahousse L, Tiemeier H, Ikram MA, Brusselle GG (2015) Chronic obstructive pulmonary disease and cerebrovascular disease: A comprehensive review. Respir Med 109(11):1371–1380 26342840 10.1016/j.rmed.2015.07.014

[36] Mehta V, Vasu TS, Phillips B, Chung F (2013) Obstructive sleep apnea and oxygen therapy: A systematic review of the literature and Meta-Analysis. J Clin Sleep Med JCSM Off Publ Am Acad Sleep Med 9(3):271–279 10.5664/jcsm.2500 PMC3578679 23493498

[37] Patil SP, Ayappa IA, Caples SM, Kimoff RJ, Patel SR, Harrod CG (2019) Treatment of adult obstructive sleep apnea with positive airway pressure: an american academy of sleep medicine systematic review, meta-analysis, and GRADE assessment. J Clin Sleep Med 15(2):301–334 10.5664/jcsm.7638 PMC6374080 30736888

[38] Gottlieb DJ, Punjabi NM, Mehra R, Patel SR, Quan SF, Babineau DC et al (2014) CPAP versus oxygen in obstructive sleep apnea. N Engl J Med 370(24):2276–2285 24918372 10.1056/NEJMoa1306766 PMC4172401

[39] Wang ML, Wang C, Tuo M, Yu Y, Wang L, Yu JT et al (2020) Cognitive effects of treating obstructive sleep apnea: A Meta-Analysis of randomized controlled trials. J Alzheimers Dis JAD 75(3):705–715 32310179 10.3233/JAD-200088

[40] Lal C, Ayappa I, Ayas N, Beaudin AE, Hoyos C, Kushida CA et al (2022) The link between obstructive sleep apnea and neurocognitive impairment: an official American thoracic society workshop report. Ann Am Thorac Soc 19(8):1245–1256 35913462 10.1513/AnnalsATS.202205-380ST PMC9353960

[41] Olaithe M, Bucks RS (2013) Executive dysfunction in OSA before and after treatment: A Meta-Analysis. Sleep 36(9):1297–1305 23997362 10.5665/sleep.2950 PMC3738038

[42] D’Rozario AL, Hoyos CM, Wong KKH, Unger G, Kim JW, Vakulin A et al (2022) Improvements in cognitive function and quantitative sleep electroencephalogram in obstructive sleep apnea after six months of continuous positive airway pressure treatment. Sleep 45(6):zsac013 35029691 10.1093/sleep/zsac013 PMC9189957

[43] Aaronson JA, Hofman WF, van Bennekom CAM, van Bezeij T, van den Aardweg JG, Groet E et al (2016) Effects of continuous positive airway pressure on cognitive and functional outcome of stroke patients with obstructive sleep apnea: a randomized controlled trial. J Clin Sleep Med 12(4):533–541 10.5664/jcsm.5684 PMC4795280 26888587

[44] Stanchina ML, Welicky LM, Donat W, Lee D, Corrao W, Malhotra A (2013) Impact of CPAP use and age on mortality in patients with combined COPD and obstructive sleep apnea: the overlap syndrome. J Clin Sleep Med 9(8):767–772 10.5664/jcsm.2916 PMC3716667 23946706

[45] van Zeller M, McNicholas WT (2024) Sleep disordered breathing: OSA-COPD overlap. Expert Rev Respir Med 18(6):369–379 38932721 10.1080/17476348.2024.2373790

