# Supplementary material for: Cognitive outcomes in chronic obstructive pulmonary disease (COPD)/OSA overlap syndrome compared to obstructive sleep apnea (OSA) alone: a systematic review
Source: Sleep Breath. 2025 Sep 1;29(5):275. doi: 10.1007/s11325-025-03426-9 (PMC12402042; doi:10.1007/s11325-025-03426-9)
Supplement: Supplementary file 2 — Supplementary Material 2 [file 11325_2025_3426_MOESM2_ESM.docx]

**Cognitive outcomes in patients with COPD-OSA overlap syndrome: a systematic review.**

**Review question:**

1. Does COPD/OSA overlap syndrome have an impact on cognitive function compared to individuals with OSA alone?
2. What specific cognitive domains are most affected in individuals with COPD/OSA overlap syndrome?

**Searches:**

- First, we will use PROSPERO website to search for any on-going or completed systematic reviews with same topic, the cognitive outcomes in COPD/OSA overlap syndrome. Then, we will search digital databases using controlled vocabulary terms; keywords and medical subject headings in the following digital databases: MEDLINE (via Ovid), EMBASE (via Ovid), PsycINFO (via Ovid), CINAHL (EBSCOhost), and Cochrane Central Register of Controlled Trials (CENTRAL). The search terms for this study will include terms relating to COPD, OSA and cognitive function.

**COPD/OSA Overlap Syndrome:** COPD; Chronic Obstructive Pulmonary Disease; Obstructive Sleep Apnea; OSA; Sleep-Disordered Breathing; Overlap Syndrome. **Cognitive Function:** Global cognition; Cognition; Neuropsychology; Cognitive Functioning; Memory; Attention; Executive Function; Processing Speed; Language/fluency; Visuospatial skills. Lastly, all uploaded studies will be referenced on EndNote software, and duplicates will be removed.

**Types of studies to be included:**

- Randomized controlled trials, prospective and retrospective cohort studies which include cohort studies; case-controlled studies and cross-sectional studies.

**Inclusion Criteria:**

- Studies investigating the impact of cognitive function (such as memory, attention, executive function, processing speed, language/fluency, visuospatial skills), in COPD/OSA overlap syndrome, and OSA alone.
- There will be no restrictions for date of publication**.**

**Exclusion Criteria:**

- Non-English studies.
- Abstracts, case reports, narrative reviews, theses, books, conference proceedings, and self-reported diagnoses or physician diagnoses studies.
- Studies on non-human subjects.

# **Condition or domain being studied:**

- Chronic obstructive pulmonary disease (COPD) and obstructive sleep apnea (OSA) are common respiratory conditions that not only affect the lung function and sleep quality, but also have other complications. According to growing evidence, both COPD and OSA are associated with cognitive decline. However, both conditions in a single individual, known as COPD/OSA overlap syndrome, might lead to even more evident cognitive impairment.
- The existing research may not differentiate fully between the cognitive effects of OSA and the COPD/OSA overlap syndrome, and might not widely evaluate all relevant cognitive domains.

**Participants/Population:**

- Adult patients with confirmed diagnoses of OSA, and COPD/OSA overlap syndrome.
- Confirmed COPD diagnosis by post-bronchodilator spirometry indicating an FEV1/FVC ratio < 0.7 or an FEV1/FVC ratio below the lower limit of normal (LLN), or smoking history (>10 pack-years).
- Confirmed OSA Diagnosis via overnight oximetry, limited or full polysomnography indicating an apnea-hypopnea index (AHI) ≥ 5 and/or an oxygen desaturation index (ODI) ≥ 5.

**Intervention(s), exposure(s):**

Not applicable.

**Comparator(s)/control:**

Not applicable.

**Main outcome(s)**

- Impact of COPD-OSA overlap on cognitive function compared to individuals with OSA alone. (First research question).
- Specific cognitive domains affected. (Second research question).

**Measures of effect**

- Our measures of effect will be the mean differences, relative risks, or odds ratio depending on the availability in the retrieved studies.

**Additional outcome(s)**

**None**

**Data Selection:**

- We will be using (PRISMA-P 2015) in our review process and the studies retrieved will be imported into EndNote software for efficient organization and duplicate studies will be removed. Two independent reviewers will blindly screen titles and abstracts of all articles using Rayyan based on our including criteria (described above). A third reviewer will be consulted if there was any disagreement.

**Data Extraction:**

- General information such as title, author, and date of publication.
- The study characteristics that include objectives and aims, study design, Inclusion and exclusion criteria, participant demographic, and baseline characteristics, primary and secondary outcomes related to cognitive function.
- The criteria used to diagnose COPD/OSA overlap syndrome.

**Risk of bias (quality) assessment:**

- Reviewers will independently assess methodological quality and risk of bias using Cochrane for randomised controlled trials, and modified Newcastle-Ottawa Scale for others).

**Strategy for data synthesis**

- We will be using a narrative synthesis approach to summarize the findings from the included studies. We will assess study methodologies and the potential for bias risk of bias using Cochrane or modified Newcastle-Ottawa Scale. Then we will be comparing and analysing the similarities and differences between the studies according to the specific cognitive function domains assessed. Next, we will tabulate the findings to present the measure of effects (described above) to compare the cognitive function with the overlap syndrome with those only OSA. If there was a sufficient similarity in the main outcomes, a meta-analysis will be conducted to combine these findings and estimate the overall effects of COPD/OSA overlap syndrome on cognitive function. We will lastly tabulate the key findings for each cognitive function domain comparing COPD-OSA patients with OSA alone.
