# Supplementary material for: Cognitive outcomes in chronic obstructive pulmonary disease (COPD)/OSA overlap syndrome compared to obstructive sleep apnea (OSA) alone: a systematic review
Source: Sleep Breath. 2025 Sep 1;29(5):275. doi: 10.1007/s11325-025-03426-9 (PMC12402042; doi:10.1007/s11325-025-03426-9)
Supplement: Supplementary file 3 — Supplementary Material 3 [file 11325_2025_3426_MOESM3_ESM.docx]

Embase <1974 to 2024 June 13>

Search Strategy:

| # | Searches | Results |
| --- | --- | --- |
| 1 | (COAD or COBD or COPD).mp. [mp=title, abstract, heading word, drug trade name, original title, device manufacturer, drug manufacturer, device trade name, keyword heading word, floating subheading word, candidate term word] | 120045 |
| 2 | Chronic Obstructive Pulmonary Disease.mp. [mp=title, abstract, heading word, drug trade name, original title, device manufacturer, drug manufacturer, device trade name, keyword heading word, floating subheading word, candidate term word] | 95651 |
| 3 | exp Bronchitis/ | 70880 |
| 4 | exp emphysema/ | 56667 |
| 5 | pulmonary disease, chronic obstructive/ or bronchitis, chronic/ or pulmonary emphysema/ | 109662 |
| 6 | Pulmonary Disease, Chronic Obstructive.mp. [mp=title, abstract, heading word, drug trade name, original title, device manufacturer, drug manufacturer, device trade name, keyword heading word, floating subheading word, candidate term word] | 1748 |
| 7 | (lung* or pulmon* or respirat* or bronchopulmon*).mp. [mp=title, abstract, heading word, drug trade name, original title, device manufacturer, drug manufacturer, device trade name, keyword heading word, floating subheading word, candidate term word] | 3267754 |
| 8 | (COPD and OSA overlap syndrome).mp. [mp=title, abstract, heading word, drug trade name, original title, device manufacturer, drug manufacturer, device trade name, keyword heading word, floating subheading word, candidate term word] | 73 |
| 9 | Overlap syndrome.mp. [mp=title, abstract, heading word, drug trade name, original title, device manufacturer, drug manufacturer, device trade name, keyword heading word, floating subheading word, candidate term word] | 5651 |
| 10 | (overlap* adj2 syndrome*).mp. [mp=title, abstract, heading word, drug trade name, original title, device manufacturer, drug manufacturer, device trade name, keyword heading word, floating subheading word, candidate term word] | 8308 |
| 11 | Overlap* syndrome.mp. [mp=title, abstract, heading word, drug trade name, original title, device manufacturer, drug manufacturer, device trade name, keyword heading word, floating subheading word, candidate term word] | 5885 |
| 12 | overlap*.mp. [mp=title, abstract, heading word, drug trade name, original title, device manufacturer, drug manufacturer, device trade name, keyword heading word, floating subheading word, candidate term word] | 288328 |
| 13 | (OSA or OSAS).mp. [mp=title, abstract, heading word, drug trade name, original title, device manufacturer, drug manufacturer, device trade name, keyword heading word, floating subheading word, candidate term word] | 47470 |
| 14 | exp Apnea/ | 44705 |
| 15 | (apnea* or apnoea*).mp. [mp=title, abstract, heading word, drug trade name, original title, device manufacturer, drug manufacturer, device trade name, keyword heading word, floating subheading word, candidate term word] | 128143 |
| 16 | Obstructive Sleep Apnea.mp. [mp=title, abstract, heading word, drug trade name, original title, device manufacturer, drug manufacturer, device trade name, keyword heading word, floating subheading word, candidate term word] | 55841 |
| 17 | Sleep Apnea, Obstructive/ | 6188 |
| 18 | (sleep* adj3 (apnea* or apnoea*)).mp. [mp=title, abstract, heading word, drug trade name, original title, device manufacturer, drug manufacturer, device trade name, keyword heading word, floating subheading word, candidate term word] | 96510 |
| 19 | "COPD and OSA".mp. [mp=title, abstract, heading word, drug trade name, original title, device manufacturer, drug manufacturer, device trade name, keyword heading word, floating subheading word, candidate term word] | 313 |
| 20 | (COPD and OSA).mp. [mp=title, abstract, heading word, drug trade name, original title, device manufacturer, drug manufacturer, device trade name, keyword heading word, floating subheading word, candidate term word] | 1162 |
| 21 | Cognitive outcome*.mp. [mp=title, abstract, heading word, drug trade name, original title, device manufacturer, drug manufacturer, device trade name, keyword heading word, floating subheading word, candidate term word] | 10135 |
| 22 | Global cognition.mp. [mp=title, abstract, heading word, drug trade name, original title, device manufacturer, drug manufacturer, device trade name, keyword heading word, floating subheading word, candidate term word] | 3938 |
| 23 | Cognitive Function*.mp. [mp=title, abstract, heading word, drug trade name, original title, device manufacturer, drug manufacturer, device trade name, keyword heading word, floating subheading word, candidate term word] | 132468 |
| 24 | Cognitive impairment.mp. [mp=title, abstract, heading word, drug trade name, original title, device manufacturer, drug manufacturer, device trade name, keyword heading word, floating subheading word, candidate term word] | 146573 |
| 25 | (cognition or processing speed or executive function or memory or mental recall or recognition, psychology).mp. [mp=title, abstract, heading word, drug trade name, original title, device manufacturer, drug manufacturer, device trade name, keyword heading word, floating subheading word, candidate term word] | 841235 |
| 26 | ((Cognitive or Neurocognitive or Neuropsychological) adj3 (Outcome* or Decline or Function or Assessment or Test*)).mp. [mp=title, abstract, heading word, drug trade name, original title, device manufacturer, drug manufacturer, device trade name, keyword heading word, floating subheading word, candidate term word] | 264438 |
| 27 | Mental*.mp. [mp=title, abstract, heading word, drug trade name, original title, device manufacturer, drug manufacturer, device trade name, keyword heading word, floating subheading word, candidate term word] | 1156213 |
| 28 | 1 or 2 or 3 or 4 or 5 or 6 or 7 or 9 or 10 or 11 or 12 | 3565463 |
| 29 | 13 or 14 or 15 or 16 or 17 or 18 or 19 or 20 | 135647 |
| 30 | 28 and 29 | 54301 |
| 31 | 21 or 22 or 23 or 24 or 25 or 26 or 27 | 1968754 |
| 32 | 30 and 31 | 2781 |
| 33 | (randomised or randomized or RCT or cohort or case-control or cross-sectional or observational).mp. [mp=title, abstract, heading word, drug trade name, original title, device manufacturer, drug manufacturer, device trade name, keyword heading word, floating subheading word, candidate term word] | 4556226 |
| 34 | 32 and 33 | 641 |
| 35 | exp Children/ or Children.mp. [mp=title, abstract, heading word, drug trade name, original title, device manufacturer, drug manufacturer, device trade name, keyword heading word, floating subheading word, candidate term word] | 3544072 |
| 36 | 34 not 35 | 497 |
| 37 | exp infant/ or infant.mp. [mp=title, abstract, heading word, drug trade name, original title, device manufacturer, drug manufacturer, device trade name, keyword heading word, floating subheading word, candidate term word] | 1272958 |
| 38 | 36 not 37 | 494 |
| 39 | limit 38 to english language | 486 |
