# Supplementary material for: Cognitive outcomes in chronic obstructive pulmonary disease (COPD)/OSA overlap syndrome compared to obstructive sleep apnea (OSA) alone: a systematic review
Source: Sleep Breath. 2025 Sep 1;29(5):275. doi: 10.1007/s11325-025-03426-9 (PMC12402042; doi:10.1007/s11325-025-03426-9)
Supplement: Supplementary file 5 — Supplementary Material 5 [file 11325_2025_3426_MOESM5_ESM.pdf]

Search History

#	Query	Limiters/Expanders	Last Run Via	Results
S11	S9 NOT S10	Expanders - Apply equivalent subjects<br>Search modes - Boolean/Phrase	Interface - EBSCOhost Research Databases<br>Search Screen - Advanced Search<br>Database - CINAHL Plus	43
S10	infant	Expanders - Apply equivalent subjects<br>Search modes - Boolean/Phrase	Interface - EBSCOhost Research Databases<br>Search Screen - Advanced Search<br>Database - CINAHL Plus	340,788
S9	S7 NOT S8	Expanders - Apply equivalent subjects<br>Search modes - Boolean/Phrase	Interface - EBSCOhost Research Databases<br>Search Screen - Advanced Search<br>Database - CINAHL Plus	45
S8	children OR child	Expanders - Apply equivalent subjects<br>Search modes - Boolean/Phrase	Interface - EBSCOhost Research Databases<br>Search Screen - Advanced Search<br>Database - CINAHL Plus	845,226
S7	S5 AND S6	Expanders - Apply equivalent subjects<br>Search modes - Boolean/Phrase	Interface - EBSCOhost Research Databases<br>Search Screen - Advanced Search<br>Database - CINAHL Plus	55
S6	randomised OR randomized OR RCT OR cohort OR case-control OR cross-sectional OR observational	Expanders - Apply equivalent subjects<br>Search modes - Boolean/Phrase	Interface - EBSCOhost Research Databases<br>Search Screen - Advanced Search<br>Database - CINAHL Plus	1,102,713
S5	S3 AND S4	Expanders - Apply equivalent subjects<br>Search modes - Boolean/Phrase	Interface - EBSCOhost Research Databases<br>Search Screen - Advanced Search<br>Database - CINAHL Plus	231
S4	S1 AND S2	Expanders - Apply equivalent subjects<br>Search modes - Boolean/Phrase	Interface - EBSCOhost Research Databases<br>Search Screen - Advanced Search<br>Database - CINAHL Plus	5,795
S3	"Cognitive outcome*" OR "Global cognition" OR "Cognitive Function*" OR "Cognitive impairment" OR cognition OR "processing speed" OR "executive function" OR memory OR "mental recall" OR "recognition, psychology" OR "Cognitive Outcome" OR "Neurocognitive Outcome" OR "Neuropsychological Outcome" OR Mental*	Expanders - Apply equivalent subjects<br>Search modes - Boolean/Phrase	Interface - EBSCOhost Research Databases<br>Search Screen - Advanced Search<br>Database - CINAHL Plus	507,912
S2	OSA OR OSAS OR "Sleep Apnea, Obstructive" OR apnea* OR apnoea* OR "Obstructive Sleep Apnea" OR sleep* adj3 (apnea* OR apnoea*)	Expanders - Apply equivalent subjects<br>Search modes - Boolean/Phrase	Interface - EBSCOhost Research Databases<br>Search Screen - Advanced Search<br>Database - CINAHL Plus	24,536
S1	COAD OR COBD OR COPD OR "Chronic Obstructive Pulmonary Disease" OR Bronchitis OR Emphysema OR "Pulmonary Disease, Chronic Obstructive" OR "Chronic Bronchitis" OR "Pulmonary Emphysema" OR lung* OR pulmon* OR respirat* OR bronchopulmon* OR "COPD and OSA overlap syndrome" OR "Overlap syndrome" OR overlap* adj2 syndrome* OR Overlap*	Expanders - Apply equivalent subjects<br>Search modes - Boolean/Phrase	Interface - EBSCOhost Research Databases<br>Search Screen - Advanced Search<br>Database - CINAHL Plus	436,765
