# Supplementary material for: Cognitive outcomes in chronic obstructive pulmonary disease (COPD)/OSA overlap syndrome compared to obstructive sleep apnea (OSA) alone: a systematic review
Source: Sleep Breath. 2025 Sep 1;29(5):275. doi: 10.1007/s11325-025-03426-9 (PMC12402042; doi:10.1007/s11325-025-03426-9)
Supplement: Supplementary file 6 — Supplementary Material 6 [file 11325_2025_3426_MOESM6_ESM.pdf]

Search Name: Cognitive outcomes
Last Saved: 19/06/2024 11:40:53
Comment:

ID	Search
#1	(COAD OR COBD OR COPD OR "Chronic Obstructive Pulmonary Disease" OR Bronchitis OR Emphysema OR "Pulmonary Disease, Chronic Obstructive" OR "Chronic Bronchitis" OR "Pulmonary Emphysema" OR lung* OR pulmon* OR respirat* OR bronchopulmon* OR "COPD and OSA overlap syndrome" OR "Overlap syndrome" OR overlap* adj2 syndrome* OR Overlap*):ti,ab,kw (Word variations have been searched)
#2	OSA OR OSAS OR "Sleep Apnea, Obstructive" OR apnea* OR apnoea* OR "Obstructive Sleep Apnea" OR sleep* adj3 (apnea* OR apnoea*)
#3	(Cognitive NEXT outcome*) OR Global cognition OR (Cognitive NEXT Function*) OR Cognitive impairment OR cognition OR processing speed OR executive function OR memory OR mental recall OR recognition psychology OR Cognitive Outcome OR Neurocognitive Outcome OR Neuropsychological Outcome OR Mental*
#4	#1 AND #2
#5	#3 AND #4 in Trials
#6	child* OR children
#7	#5 NOT #6
#8	Infant*
#9	#7 NOT #8
