# Supplementary material for: Cognitive outcomes in chronic obstructive pulmonary disease (COPD)/OSA overlap syndrome compared to obstructive sleep apnea (OSA) alone: a systematic review
Source: Sleep Breath. 2025 Sep 1;29(5):275. doi: 10.1007/s11325-025-03426-9 (PMC12402042; doi:10.1007/s11325-025-03426-9)
Supplement: Supplementary file 7 — Supplementary Material 7 [file 11325_2025_3426_MOESM7_ESM.pdf]

APA PsycInfo <1806 to February 2025 Week 2>

1 (COAD or COBD or COPD).mp. [mp=title, abstract, heading word, table of contents, key concepts, original title, tests & measures, mesh word] 2181

2 Chronic Obstructive Pulmonary Disease.mp. [mp=title, abstract, heading word, table of contents, key concepts, original title, tests & measures, mesh word] 3038

3 exp Bronchitis/ 0

4 exp emphysema/ 88

5 pulmonary disease, chronic obstructive/ or bronchitis, chronic/ or pulmonary emphysema/ 88

6 Pulmonary Disease, Chronic Obstructive.mp. [mp=title, abstract, heading word, table of contents, key concepts, original title, tests & measures, mesh word] 1170

7 (lung* or pulmon* or respirat* or bronchopulmon*).mp. [mp=title, abstract, heading word, table of contents, key concepts, original title, tests & measures, mesh word] 46292

8 (COPD and OSA overlap syndrome).mp. [mp=title, abstract, heading word, table of contents, key concepts, original title, tests & measures, mesh word] 1

9 Overlap syndrome.mp. [mp=title, abstract, heading word, table of contents, key concepts, original title, tests & measures, mesh word] 70

10 (overlap* adj2 syndrome*).mp. [mp=title, abstract, heading word, table of contents, key concepts, original title, tests & measures, mesh word] 234

11 Overlap* syndrome.mp. [mp=title, abstract, heading word, table of contents, key concepts, original title, tests & measures, mesh word] 75

12 overlap*.mp. [mp=title, abstract, heading word, table of contents, key concepts, original title, tests & measures, mesh word] 50109

13 (OSA or OSAS).mp. [mp=title, abstract, heading word, table of contents, key concepts, original title, tests & measures, mesh word] 3055

14 exp Apnea/ 4551

15 (apnea* or apnoea*).mp. [mp=title, abstract, heading word, table of contents, key concepts, original title, tests & measures, mesh word] 7859

16 Obstructive Sleep Apnea.mp. [mp=title, abstract, heading word, table of contents, key concepts, original title, tests & measures, mesh word] 3979

17 Sleep Apnea, Obstructive/ 0

18 (sleep* adj3 (apnea* or apnoea*)).mp. [mp=title, abstract, heading word, table of contents, key concepts, original title, tests & measures, mesh word] 6788

19 "COPD and OSA".mp. [mp=title, abstract, heading word, table of contents, key concepts, original title, tests & measures, mesh word] 6

20 (COPD and OSA).mp. [mp=title, abstract, heading word, table of contents, key concepts, original title, tests & measures, mesh word] 22

21 Cognitive outcome*.mp. [mp=title, abstract, heading word, table of contents, key concepts, original title, tests & measures, mesh word] 4303

22 Global cognition.mp. [mp=title, abstract, heading word, table of contents, key concepts, original title, tests & measures, mesh word] 1747

23 Cognitive Function*.mp. [mp=title, abstract, heading word, table of contents, key concepts, original title, tests & measures, mesh word] 55918

24 Cognitive impairment.mp. [mp=title, abstract, heading word, table of contents, key concepts, original title, tests & measures, mesh word] 69980

25 (cognition or processing speed or executive function or memory or mental recall or recognition, psychology).mp. [mp=title, abstract, heading word, table of contents, key concepts, original title, tests & measures, mesh word] 466528

26 ((Cognitive or Neurocognitive or Neuropsychological) adj3 (Outcome* or Decline or Function or Assessment or Test*)).mp. [mp=title, abstract, heading word, table of contents, key concepts, original title, tests & measures, mesh word] 158469

27 Mental*.mp. [mp=title, abstract, heading word, table of contents, key concepts, original title, tests & measures, mesh word] 749663

28 1 or 2 or 3 or 4 or 5 or 6 or 7 or 9 or 10 or 11 or 12 96188

29 13 or 14 or 15 or 16 or 17 or 18 or 19 or 20 8117

30 28 and 29 2488

31 21 or 22 or 23 or 24 or 25 or 26 or 27 1190964

32 30 and 31 404

33 (randomised or randomized or RCT or cohort or case-control or cross-sectional or observational).mp. [mp=title, abstract, heading word, table of contents, key concepts, original title, tests & measures, mesh word] 431930

34 32 and 33 104

35 exp Children/ or Children.mp. [mp=title, abstract, heading word, table of contents, key concepts, original title, tests & measures, mesh word] 624887

36 34 not 35 80

37 exp infant/ or infant.mp. [mp=title, abstract, heading word, table of contents, key concepts, original title, tests & measures, mesh word] 100194

38 36 not 37 78

39 limit 38 to english language 77

40 (COAD or COBD or COPD).mp. [mp=title, abstract, heading word, table of contents, key concepts, original title, tests & measures, mesh word] 2181

41 Chronic Obstructive Pulmonary Disease.mp. [mp=title, abstract, heading word, table of contents, key concepts, original title, tests & measures, mesh word] 3038

42 exp Bronchitis/ 0

43 exp emphysema/ 88

44 pulmonary disease, chronic obstructive/ or bronchitis, chronic/ or pulmonary emphysema/ 88

45 Pulmonary Disease, Chronic Obstructive.mp. [mp=title, abstract, heading word, table of contents, key concepts, original title, tests & measures, mesh word] 1170

46 (lung* or pulmon* or respirat* or bronchopulmon*).mp. [mp=title, abstract, heading word, table of contents, key concepts, original title, tests & measures, mesh word] 46292

47 (COPD and OSA overlap syndrome).mp. [mp=title, abstract, heading word, table of contents, key concepts, original title, tests & measures, mesh word] 1

48 Overlap syndrome.mp. [mp=title, abstract, heading word, table of contents, key concepts, original title, tests & measures, mesh word] 70

49 (overlap* adj2 syndrome*).mp. [mp=title, abstract, heading word, table of contents, key concepts, original title, tests & measures, mesh word] 234

50 Overlap* syndrome.mp. [mp=title, abstract, heading word, table of contents, key concepts, original title, tests & measures, mesh word] 75

51 overlap*.mp. [mp=title, abstract, heading word, table of contents, key concepts, original title, tests & measures, mesh word] 50109

52 (OSA or OSAS).mp. [mp=title, abstract, heading word, table of contents, key concepts, original title, tests & measures, mesh word] 3055

53 exp Apnea/ 4551

54 (apnea* or apnoea*).mp. [mp=title, abstract, heading word, table of contents, key concepts, original title, tests & measures, mesh word] 7859

55 Obstructive Sleep Apnea.mp. [mp=title, abstract, heading word, table of contents, key concepts, original title, tests & measures, mesh word] 3979

56 Sleep Apnea, Obstructive/ 0

57 (sleep* adj3 (apnea* or apnoea*)).mp. [mp=title, abstract, heading word, table of contents, key concepts, original title, tests & measures, mesh word] 6788

58 "COPD and OSA".mp. [mp=title, abstract, heading word, table of contents, key concepts, original title, tests & measures, mesh word] 6

59 (COPD and OSA).mp. [mp=title, abstract, heading word, table of contents, key concepts, original title, tests & measures, mesh word] 22

60 Cognitive outcome*.mp. [mp=title, abstract, heading word, table of contents, key concepts, original title, tests & measures, mesh word] 4303

61 Global cognition.mp. [mp=title, abstract, heading word, table of contents, key concepts, original title, tests & measures, mesh word] 1747

62 Cognitive Function*.mp. [mp=title, abstract, heading word, table of contents, key concepts, original title, tests & measures, mesh word] 55918

63 Cognitive impairment.mp. [mp=title, abstract, heading word, table of contents, key concepts, original title, tests & measures, mesh word] 69980

64 (cognition or processing speed or executive function or memory or mental recall or recognition, psychology).mp. [mp=title, abstract, heading word, table of contents, key concepts, original title, tests & measures, mesh word] 466528

65 ((Cognitive or Neurocognitive or Neuropsychological) adj3 (Outcome* or Decline or Function or Assessment or Test*)).mp. [mp=title, abstract, heading word, table of contents, key concepts, original title, tests & measures, mesh word] 158469

66 Mental*.mp. [mp=title, abstract, heading word, table of contents, key concepts, original title, tests & measures, mesh word] 749663

67 40 or 41 or 42 or 43 or 44 or 45 or 46 or 48 or 49 or 50 or 51 96188

68 52 or 53 or 54 or 55 or 56 or 57 or 58 or 59 8117

69 67 and 68 2488

70 60 or 61 or 62 or 63 or 64 or 65 or 66 1190964

71 69 and 70 404

72 (randomised or randomized or RCT or cohort or case-control or cross-sectional or observational).mp. [mp=title, abstract, heading word, table of contents, key concepts, original title, tests & measures, mesh word] 431930

73 71 and 72 104

74 exp Children/ or Children.mp. [mp=title, abstract, heading word, table of contents, key concepts, original title, tests & measures, mesh word] 624887

75 73 not 74 80

76 exp infant/ or infant.mp. [mp=title, abstract, heading word, table of contents, key concepts, original title, tests & measures, mesh word] 100194

77 75 not 76 78

78 limit 77 to english language 77
